# Prevalence of hepatitis B among childbearing women and infant born to HBV-positive mothers in Togo

**DOI:** 10.1186/s12879-020-05574-7

**Published:** 2020-11-12

**Authors:** Didier K. Ekouevi, Lucile Larrouy, Fifonsi A. Gbeasor-Komlanvi, Vincent Mackiewicz, Martin K. Tchankoni, Alexandra M. Bitty-Anderson, Gatibe Yendu-suglpak Gnatou, Arnold Sadio, Mounerou Salou, Claver A. Dagnra, Diane Descamps, Patrick A. Coffie

**Affiliations:** 1 Département de Santé Publique, Faculté des Sciences de la Santé, Université de Lomé, Lomé, Togo; 2 Institut de Santé Publique Epidémiologie Développement (ISPED), Université de Bordeaux, Bordeaux, France; 3 Programme PACCI – Site ANRS Côte d’Ivoire, CHU de Treichville, Abidjan, Côte d’Ivoire; 4 INSERM U1219 Bordeaux Population Health Research, ISPED, Université de Bordeaux, Bordeaux, France; 5 Centre Africain de Recherche en Epidémiologie et en Santé Publique (CARESP), Lomé, Togo; 6 Université de Paris, INSERM UMR 1137 IAME, F-75018 Paris, France; 7 Laboratoire de Virologie, AP-HP, Hôpital Bichat-Claude Bernard, F-75018 Paris, France; 8 Département des Sciences Fondamentales, Laboratoire de Biologie Moléculaire, Université de Lomé, Lomé, Togo; 9 Programme National de Lutte contre le VIH/Sida, les Hépatites virales et les Infections Sexuellement Transmissibles (PNLS/HV/IST), Lomé, Togo; 10 Département de Dermatologie et d’Infectiologie, UFR des Sciences Médicales, Université Félix Houphouët Boigny, Abidjan, Côte d’Ivoire; 11 Service des Maladies Infectieuses et Tropicales, Centre Hospitalier Universitaire de Treichville, Abidjan, Côte d’Ivoire

**Keywords:** Hepatitis B, Childbearing-women, Infants, Vaccination, Togo

## Abstract

**Background:**

Hepatitis B virus (HBV) infection is a public health problem in Togo and transmission to the child occurs mainly during childbirth. The objective of this study was to estimate the prevalence of HBV among childbearing women and infants born to HBV positive mothers in Togo.

**Methods:**

A national cross-sectional study was carried out in six cities in Togo in the six health regions in Togo. Mother-child pairs were recruited from immunization centers or pediatric wards in Lomé, Tsévié, Atakpamé, Sokodé, Kara and Dapaong in 2017. Women aged 18 and over with one child of at least 6 months old were included. A standardized questionnaire was used for data collection and HBV screening was performed using Determine® rapid tests. The prevalence of HBV, defined by a positive HBV surface antigen (HBsAg), was estimated in mothers and then in infants of mothers who were positive for HBsAg. Logistic regression model was performed to identify risk factors for HBsAg positivity in mothers.

**Results:**

A total of 2105 mothers-pairs child were recruited. The median age of mothers and infants was 29 years, interquartile range (IQR) [25–33] and 2.1 years, IQR [1–3] respectively. About 35% of women were screened for HBV during antenatal care and 85% of infants received three doses of HBV immunization. Among mothers, the prevalence of HBV was 10.6, 95% confidence interval (95% CI) [9.4–12.0%], and 177 had detectable HBV viral load (> 10 IU/mL). Among mothers with positive HBsAg, three infants also had positive HBsAg, a prevalence of 1.3, 95% CI [0.2–3.8%]. In multivariable analysis, HIV-infection (aOR = 2.19; *p* = 0.018), having at least three pregnancies (aOR = 1.46; *p* = 0.025) and living in Tsévié (aOR = 0.31; *p* < 0.001) compared to those living in Lomé, were associated to HBV infection in mothers.

**Conclusion:**

In this study, one out of 10 childbearing women were infected with HBV, but less than 2% of infant born to HBV positive mothers under 5 years’ old who received immunization under the Expanded Program on Immunization were infected. Improving antenatal screening and providing targeted interventions in babies could help eliminate HBV in Togo.

## Background

Hepatitis B virus (HBV) infection is a global public health problem with 257 million people who are chronically infected in the world [1]. HBV prevalence is the highest in Western Pacific Regions and in Africa, both accounting for 68% of those infected worldwide, with respective prevalence of 6.2 and 6.1% [1]. Each year, around 1 million people die from HBV mostly due to cirrhosis and hepatocellular carcinoma (HCC) [2, 3]. In West Africa, approximately 8% of HIV-infected individuals have chronic HBV infection [4, 5]. In this part of the world, HBV infection is one of the main causes of liver disease, cirrhosis and HCC [4, 5].

Without effective interventions and an accelerated response, the number of people living with HBV is expected to remain at the current high levels, with a cumulative 20 million deaths between 2015 and 2030 [1]. These deaths will mainly occur in developing countries of Asia and Africa. In 2016, the United Nations General Assembly and the World Health Organization (WHO) incorporated HBV elimination in the 2030 agenda for sustainable development goals and the first global health section strategy on HBV was developed [1]. However, elimination of HBV will not be achieved without controlling perinatal transmission of HBV, which is among the five core intervention areas for the global health sector strategy on viral hepatitis 2016–2021 [1].

Most of the data on HBV mother-to-child transmission (MTCT) have been collected in Asia and interventions to prevent HBV MTCT have been implemented in most Asian countries [6–8]. The Western Pacific Region was able to achieve regional hepatitis B control through the prevention of HBV MTCT and the implementation of HBV birth dose immunization [9, 10]. In the African region, the rate and risk factors of HBV MTCT, as well as the efficacy of preventive measures including HBV birth dose immunization have been poorly assessed [9]. Due to the lack of data, the WHO is currently unable to provide strong recommendations on prevention of HBV MTCT, particularly with regard to the use of tenofovir during pregnancy [1, 11]. In order to prevent HBV MTCT, the WHO recommends a timely administration of HBV immunization at birth i.e. during the first 24 h of life in all infants born in endemic countries [2], even if the administration of HBV birth dose immunization alone is not sufficient to prevent HBV MTCT in infants born to highly viremic mothers [9]. Moreover, only 11 countries in Africa included the HBV birth dose as part of the routine infant immunization schedule in 2017 [9, 12].

In Togo, HBV immunization was introduced in the Expanded Program of Immunization (EPI) in 2008 and infants receive HBV immunization at six, 10 and 14 weeks of life. In this country where HBV birth dose is not yet endorsed by the EPI, little is known about the prevalence of HBV among infants exposed to HBV. In adults, HBV prevalence was estimated at 9% among people living with HIV (PLHIV) in 2011 and 7% among men who have sex with men in 2017 [13]. The objective of this study was to estimate the prevalence of hepatitis B among mother and infants born to HBV positive mothers in Togo.

## Methods

### Study design and settings

This study was a cross-sectional study conducted from August to September 2017 in Togo. Togo is a country of West Africa which covers an area of 56,800 Km^2^ with an average density of 145 inhabitants per square kilometer. The population was 7.89 million in 2018, of which 50.2% are women. Most of the population is young (60% of Togolese are under 25 years of age), and lives in rural areas (62%). In Togo, testing for HBsAg is mandatory but must be paid by mothers and in case of HBsAg positive, only the immunization at the birth dose is recommended, but it must also be paid for by the mothers. Also, tenofovir is not recommended.

### Population and sample size calculation

In Togo, there is a consultation for infants under 5 years of age where the weight and height (growth) of the infants are systematically checked. It is carried out at the time of vaccination of infants under 2 years of age and in paediatric wards between two and 5 years of age. For the selection of the population: (i) we conducted the survey in all of the six health regions in Togo, in the main city of the region; (ii) secondly, we randomly selected in each health regions one or two facilities with vaccination center and with a laboratory available in urban city; (iii) finally a consecutively sampled method was used to select eligible participants. All mothers above 18 years with infants aged between 6 months and 5 years’ old who attended under 5 years’ consultation in selected health structures during the survey period were invited to participate in the study. The health card of recruited infants was marked with a sticker to avoid double counting. The sample size to estimate the prevalence of HBV in mothers was calculated using a single proportion population formula with a 95% confidence level, 4% margin of error and 10% estimated prevalence of HBV among women in Togo [14]. A 10% non-response rate was considered and the minimum number of mother-infant couples for each health region was estimated at 238. We therefore included at least 300 mother-infant couples in each health region.

### Study procedures and data collection

After eligibility screening and informed consent approval, a standardized questionnaire was administered by trained last year medical students during a face-to-face interview. Data collected included information on socio-demographic characteristics, risky behaviors, immunization coverage, HBV and HIV testing history, access to treatment care services. The information on immunization were collected from immunization card and based on recall of injection site. The information at birth dose vaccination in infant born to HBsAg positive mothers in each main city of six health regions, such as HIV and HBV testing were collected in antenatal care card. When a mother brought more than one children under 5 years, the youngest was included.

### Laboratory testing

Venous blood samples were collected in mothers and their infants. However only infant from HBsAg positive mothers were tested for HBV. They were tested in the laboratory reference center of the main hospital in selected cities for HBV infection using a validated rapid point-of-care (POC) for HBs antigen (HBsAg) detection (Alere Determine™ HBsAg, Abbott, Chicago, USA) [15, 16]. Pre and post HBV test counselling were given, and HBV status was given the following date on clinical site. The remaining blood samples were sent to the national biological reference center “Laboratoire de Biologie Moléculaire (BIOLIM)” (Molecular biology laboratory), Université of Lomé. The aliquots were then sent to the Virology Laboratory of Bichat-Claude Bernard Hospital in Paris for HBV DNA.

Samples reactive for HBsAg in mothers and infants were subsequently tested for HBV DNA. HBV DNA viral load was quantified in plasma with Cobas HBV® test on a 6800 system (Roche Diagnostics GmbH.). Viral nucleic acid was extracted from plasma and HBV DNA was amplified by real-time PCR with a detection limit of 10 IU/mL. This molecular assay was performed according to manufacturer’s instructions, in the ISO 15189 accredited Virology Laboratory of Bichat-Claude Bernard Hospital in Paris.

### Statistical analysis

Descriptive analyses were performed, and results were presented with frequency and proportions. Prevalence rates were estimated with their 95% confidence interval (95% CI). Continuous variables were described with median and interquartile range (IQR). Groups’ comparisons were performed using Student’s t-test or non-parametric Wilcoxon rank-sum test (non-normal distribution) for continuous variables and using Chi-2 test or Fisher’s exact test for categorical variables. Univariable and multivariable logistic regression analyses were performed with a stepwise-descending selection procedure to identify factors associated with HBsAg positivity in mothers. The selection of covariates for multivariable analysis was based on the univariable analyses with factors associated with HBsAg positivity (*p* < 0.25). Adjusted Odds Ratios (aORs) were reported with corresponding 95%CI. We deemed a *p*-value < 0.05 as statistically significant for all analyses. Data analyses were performed using Stata software (Stata™ 11.0 College Station, Texas, USA).

### Ethical consideration

This study was approved by the “Comité de Bioéthique pour la Recherche en Santé (CBRS)” (Bioethics Committee for Health Research) from the Ministry of Health of Togo. Potential participants were told about the study purpose and procedures, potential risks and protections, and compensation. Informed consent was documented with signed consent forms prior to participation in the study and informed consent from mothers was also required for the blood sample collection from their children.

## Results

### Socio-demographic and clinical characteristics of mothers and infants

A total of 2105 mother-infant couples were enrolled in six cities in Togo. The median age of mothers was 29 years, IQR [25–33], 73.3% (*n* = 1542) were able to write and read and 94.8% (*n* = 1995) were married or were in relationship. Socio-demographic characteristics are summarized in Table 1. In regard to infants, their median age was 27 months, IQR [14–41], 50.9% (*n* = 1072) were boys and 84.3% (*n* = 1774) received at least three doses of HBV immunization (Table 2).

**Table 1:** Sociodemographic characteristics of mothers (*N* = 2105)

	Frequency	Proportion (%)
**Age (years) (*N*** **= 2105)**		
**Median**	29	IQR [25–33]
< 25	514	24.4
[25–30[	648	30.8
≥ 30	943	44.8
**City (N = 2105)**		
Lome	340	16.2
Atakpame	312	14.8
Dapaong	361	17.1
Kara	353	16.8
Sokode	379	18.0
Tsevie	360	17.1
**Able to write and read (N = 2105)**		
No	563	26.7
Yes	1542	73.3
**Marital status (N = 2105)**		
Single	79	3.7
Married/In relationship	1995	94.8
Divorced/Widow	31	1.5
**Number of pregnancies (N = 2105)**		
1	527	25.0
2	568	27.0
3	432	20.5
≥ 4	578	27.5
**Number of deliveries (N = 2105)**		
1	688	32.7
2	579	27.5
≥ 3	838	39.8
**History of HIV testing (*N*** **= 2051)**		
No	644	30.6
Yes^a^	1459	69.4
**HIV status (*N*** **= 1459)**		
Negative	1382	94.7
Positive	77	5.3
**History of HBV testing (N = 1459)**		
No	715	49.0
Yes	744	51.0
**HBV status during last pregnancy (*N*** **= 744)**		
Negative	643	86.4
Positive	101	13.6
**Number of antenatal consultation (N = 2105)**		
< 4	588	27.9
≥ 4	1517	72.1
**Have heard about HBV (N = 2105)**		
No	923	43.8
Yes	1182	56.2

*HBV* hepatitis B virus, *IQR* interquartile range, ^a^Mother-child pairs health card available

**Table 2:** Characteristics of children (*N* = 2105)

	Frequency	Proportion (%)
**Age (years)**		
< 1	529	25.1
≥ 1	1576	74.9
**Sex**		
Male	1072	50.9
Female	1033	49.1
**Immunization coverage**^**a**^		
**week 6**	1844	87.6
**week 10**	1833	87.1
**week 14**	1789	85.0
**Coverage 3 doses of immunization**		
Yes	1774	84.3
No	105	5.0
Not available	226	10.7
**History of scarification**		
Yes	554	26.3
No	1551	73.7

^**a**^DTCoq Polio-HBV

### HIV and HBV history testing in antenatal care

Data recorded from maternal health card or immunization card show that 1459 (69.4%) mothers had performed HIV testing and among them, 5.4% were HIV-infected. Also, 740 (35.1%) women had performed HBV test during antenatal consultation and 13.6% were HBsAg-positive.

### Prevalence of HBV in mothers and their infants and associated factors

**Fig. 1:**
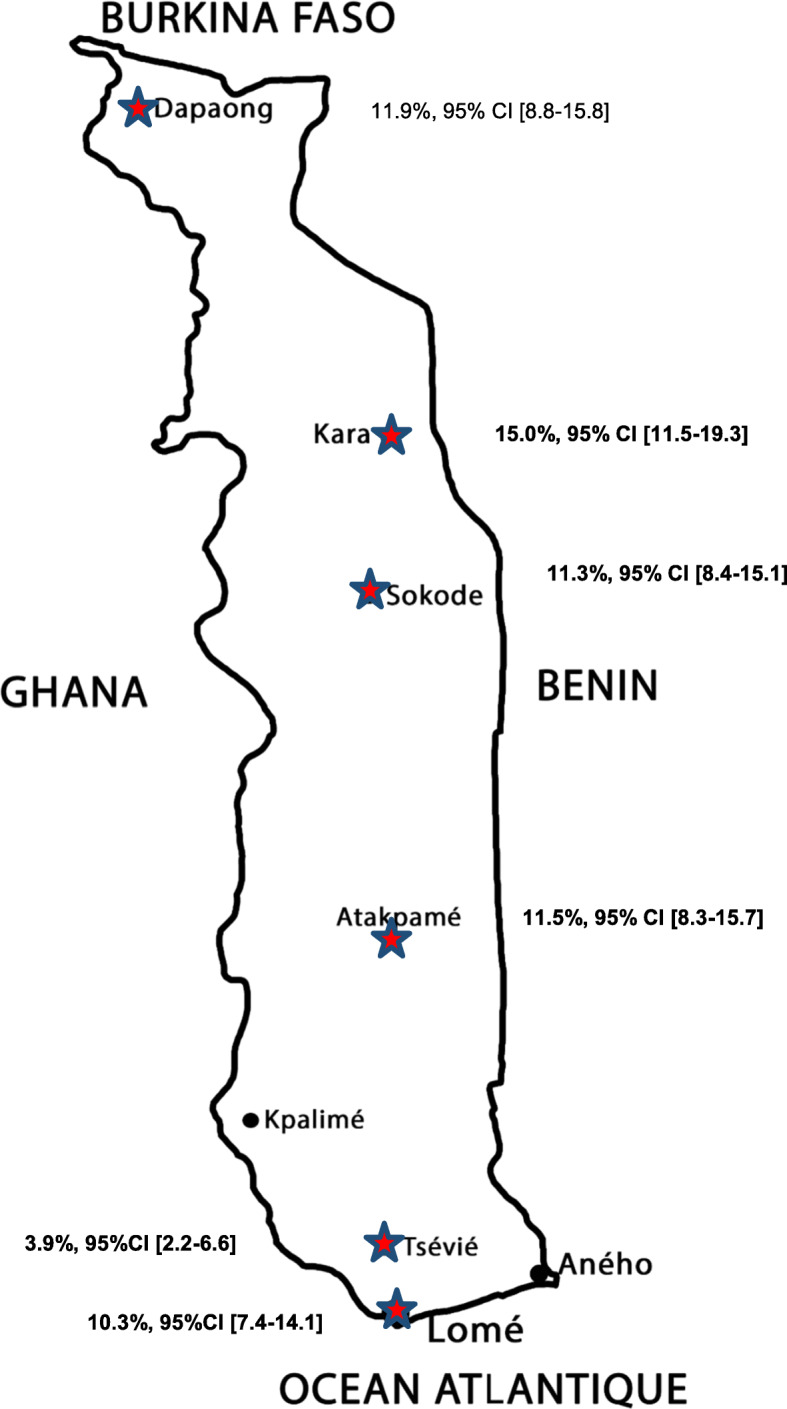
Prevalence of hepatitis B among women of childbearing age, Togo, 2017. This figure shows the prevalence of HBV infection among women of childbearing age in six cities in Togo, from south to north: Lomé, Tsévié, Atakpame, Sokode, Kara, Dapaong. The map was elaborated by ourselves, using Adobe Photoshop CC 2019 software version 20.0.4 by Adobe Systems Incorporated**©** (https://www.adobe.com/fr/products/photoshop.html#)

A total of 224 women were diagnosed with hepatitis B, yielding a prevalence of 10.6, 95% CI [9.4–12.0%]. Figure 1 shows the prevalence of hepatitis B according to the cities.

The prevalence of hepatitis B was above 10% in all of the six cities except in one city in the South of Togo (Tsévié) where the prevalence of hepatitis B was 3.9% (*p* < 0.001). In multivariable analysis, after adjusting for age and marital status, women infected with HIV (aOR = 2.19; *p* = 0.018), and women with at least three pregnancies (aOR = 1.46; *p* = 0.025) had higher risk of HBV infection. Conversely, compared to women living in Lomé, those living in Tsévié were less likely to be infected with hepatitis B (aOR = 0.31; p < 0.001) (Table 3).

**Table 3:** Factors associated with positive HBsAg in mothers, Togo, 2017

	Univariable analysis			Multivariable analysis		
	OR	95%CI	*P*-value	aOR	95%CI	*P*-value
**Age (years)**						
< 25	1	–	–	–	–	–
[25–30[	1.01	[0.68–1.51]	0.965	0.88	[0.58–1.33]	0.527
≥ 30	1.35	[0.95–1.94]	0.099	1.92	[0.68–1.55]	0.919
**City**						
Lomé	1	–	–	1	–	–
Atakpamé	1.14	[0.69–1.87]	0.611	1.15	[0.66–1.93]	0.583
Dapaong	1.18	[0.74–1.90]	0.497	1.24	[0.75–2.06]	0.395
Kara	1.54	[0.98–2.44]	0.063	1.54	[0.97–2.45]	0.067
Sokodé	1.12	[0.70–1.80]	0.651	1.06	[0.66–1.74]	0.799
Tsévié	0.35	[0.18–0.65]	0.001	0.31	[0.16–0.58]	< 0.001
**Education level**^**a**^						
No	1	–	–	–	–	–
Yes	1.02	[0.75–1.41]	0.884	–	–	–
**Marital status**						
Single/Divorced/Widow	1	–	–	1	–	–
Married/in relationship	1.80	[0.89–4.30]	0.140	1.73	[0.84–4.17]	0.175
**Number of pregnancy**						
< 3	1	–	–	1	–	–
≥ 3	1.45	[1.10–1.92]	0.009	1.46	[1.05–2.05]	0.025
**HIV status**						
Negative	1	–	–	1	–	–
Positive	1.79	[0.92–3.24]	0.065	2.19	[1.10–4.07]	0.018
Not available	1.08	[0.80–1.45]	0.628	0.97	[0.70–1.35]	0.867

*OR* odds ratio, *aOR* adjusted odds ratio, *95%CI* 95% confidence interval; ^a^ able to write and read

### HBV viral characteristics and factors associated with detectable HBV DNA

Among the 224 HBsAg-positive women, 223 (99.5%) had a blood sample available for HBV DNA measurement. Overall, HBV DNA was detected in 177 of 223 (79.3%) with a median of 346 IU/mL, IQR [78–2950]. Eighteen (10.2%) had HBV DNA > 20,000 IU/mL, and 16 (9.0%) had HBV DNA > 1 million IU/mL. Regarding factors associated with detectable HBV DNA (*n* = 223), only HIV status of women was associated with detectable HBV DNA. Indeed, HIV positive women (aOR = 0.12; p < 0,001) as well as women with unknown HIV status (aOR = 0.39; *p* = 0.024) were less likely to have detectable HBV load (> 10 IU/mL) compared to HIV negative women (Table 4).

**Table 4:** Factors associated with detectable viral load (VL > 10 copies/mL) (*N* = 223)

	Univariable analysis			Multivariable analysis		
	OR	95%CI	P-value	OR	95%CI	P-value
**Age (years)**						
< 30	1			–		
≥ 30	0.99	0.52–1.90	0.981	–		
**City**						
South	1			1		
North	1.45	0.75–2.79	0.269	2.03	0.93–4.52	0.077
**Education level**						
No	1			–		
Yes	1.28	0.61–2.58	0.493	–		
**Number of pregnancy**						
< 3	1			–		
≥ 3	1.22	0.63–2.34	0.552	–		
**HIV status**						
Negative	1			1		
Positive	0.13	0.04–0.41	0.001	0.12	0.04–0.39	< 0.001
Not available	0.54	0.26–1.11	0.091	0.39	0.17–0.88	0.024
**Knowing HBV**						
No	1			–		
Yes	1.12	0.58–2.16	0.731	–		

*OR* odds ratio, *aOR* adjusted odds ratio, *95%CI* 95% confidence interval

### Prevalence of HBV infection in infants born to women with positive HBsAg

Three out of 224 infants of HBsAg-positive women were positive for HBsAg, resulting in a prevalence of 1.3, 95% CI [0.2–3.8%] in infants. By stratifying on mother’s HBV DNA level, the prevalence of HBsAg-positive was 0.4, 95% CI [0,01-2,6%] and 12.5, 95% CI [1.5–38.3%] in infants born to women with HBV-DNA < 1 million IU/mL and ≥ 1 million IU/mL, respectively (*p* < 0.001). Table 5 summarizes socio-demographic and clinical characteristics as well as infant immunization of the three infants with HBsAg-positive.

**Table 5:** Description of three cases of HBV among children

Number	Age (months)	Sex	Mother’s characteristics		Immunization coverage in children				Scarification
			HBV VL IU/mL	HIV status	Birth	Week-6	Week-10	Week-14	
1	36	Girl	25	NA	No	Yes	Yes	Yes	No
2	30	Girl	716,000,000	NA	No	Yes	Yes	No	No
3	28	Boy	270,000,000	NA	No	Yes	Yes	No	Yes

*HBV* hepatitis B virus, *VL* viral load, *NA* not available

## Discussion

This study is the first study which described the prevalence of HBV among 2105 women of childbearing age in a national survey in Togo showed a high prevalence of HBV estimated at 10%. The distribution of HBV viral load was also described with 10% of women with HBsAg-positive eligible for HBV treatment > 20,000 IU/mL. In addition, the prevalence of HBsAg among infants less than 5 years born from women with HBsAg+ was < 2%.

The overall prevalence of HBsAg was 10% among mothers in Togo in 2018, and it was similar to that observed in HIV positive people in 2011 in Lomé (9.7%) [17], in pregnant women from West and Central African region (7–15%) [18–22] and in men who have sex with men in 2017 7.1% in Togo [13]. In our study, the prevalence of HBsAg was similar across the different regions (10%) except in one region in the South of Togo where the prevalence was 4%. In a national study conducted among MSM in 2017 in Togo, the prevalence of HBV was low in the south, varying between 0 and 4% with an increase in this prevalence from South to North [13]. This could be explained by traditional practices such as scarification more common in the north of the country. Additional case-control studies are needed to confirm this hypothesis.

In our study, the prevalence of HBsAg in infants under five in this study was low (< 2%). This finding was in accordance with the data from West African region where the HBV prevalence in infants was 0.5% in Côte d’Ivoire in 2001–2003 [23] and 1.1% in Senegal in 2016 [24]. This low prevalence in these studies could be explained by: i) the introduction of HBV immunization in the Expanded Programme on Immunization in the 2000s in West Africa; (ii) some of the mothers tested positive for HBV during pregnancy and their babies (49/101, 48.5%) received HBV immunization at birth in our study; (iii) the low proportion of women with HBV DNA level > 20,000 IU/mL. Indeed, previous studies have found that HBV transmission is correlated with viral load levels [18, 25].

In our study, although the HBV prevalence among infants is low, it is strongly linked to the HBV viral load of the mothers. Indeed, among the 15 women with high risk transmission (HBV viral load > 10^6^ IU/mL), the prevalence in infants was high (13.3%). This raises the problem of systematic antenatal screening of HBV during pregnancy in Togo as recommended by WHO [26] and the appropriate interventions to reduce HBV MTCT [11]. In our study, only 35% of women were screened for HBV during antenatal care. The reason might be that HBV test is supported by pregnant women, in contrary to HIV test which is free of charge. There is an urgent need to endorse this test and to propose it free of charge. Also, in Togo, since 2017, the national program for HIV and sexually transmitted infections control in Togo recommends the HBV birth dose immunization in the absence of a national program for hepatitis control [27, 28]. However, there is a lack of traceability for the documentation of the implementation of this recommendation. Among mothers with very high viral loads, it would be essential to treat them with tenofovir.

Co-infection with HIV was the only factor associated with undetectable HBV viral load among women with positive HBsAg. HIV positive women with HBsAg+ were less likely to have an undetectable viral load compared to HIV negative women. With the “test and treat” approach used in HIV care and treatment, most HIV positive women received standard antiretroviral therapy containing tenofovir, a drug that is also effective against hepatitis B. This therapy has helped reduce the transmission of HBV to infants. This emphasizes the importance of an integrated approach to HIV and hepatitis screening and treatment. Peripartum antiviral treatment is part of international guidelines including the European Association for the Study of the liver (EASL) and American Association for the study of liver diseases (AASLD) but is not yet part of the WHO recommendations, although this is being discussed [29, 30].

This study has some limitations. First, women of childbearing age were recruited only in urban areas and do not allow to compare with data on rural areas. Second, the quantity of blood samples collected in infants under five did not allow to perform HBV viral load measurement in the three infants with positive HBsAg. Third, we did not perform the measurement of HBeAg, another marker of replication of hepatitis B. Fourth, some infants born to women diagnosed infected with HBV during antenatal care received HBV birth dose immunizations, as it is recommended since 2017 in Togo. However, since it is at the charge of mothers, this was not systematically reported in infants’s immunization cards and there is a lack of traceability of the HBV birth dose immunization. This could have impacted the prevalence of HBV among infants.

Despite these limitations, this study has several strengths. Indeed, this study has included a large sample size all of six regions of Togo, from North to South. To our knowledge, this is the first study in West Africa reporting data on prevalence of HBsAg among women and infants under 5 years old in the context of no HBV immunization at birth. In addition, this study is one of the first to include the description of HBV viral load among women of childbearing age and their infants to better understand the major contribution of MTCT to HBV transmission.

This study provides additional data on the debate on the two approaches toward the elimination of hepatitis B in Africa where the majority of transmission occurs at birth and in early childhood. In Togo, the current approach is the adoption of a targeted administration of birth dose immunization for only babies born to positive HBsAg women, while babies born from HBV-uninfected women receive HBV immunization at six, 10 and 14 weeks of life in the EPI [31]. Despite the cost of monovalent immunization (approximately 6 USD), which is not yet included in the immunization program, women infected with HBV are highly adherent to purchasing this immunization demonstrating the effectiveness of HBV sensitization campaigns. However, access to HBV screening, care and treatment for mothers and infants remains low, below 35%. It would be important to strengthen access to screening for pregnant women, to improve access to tenofovir and to promote the implementation of the HBV birth dose immunization [11]. Better documentation of the impact of birth dose immunization is needed in Africa. Moreover, there is urgent need to conduct clinical trial on the efficacy of tenofovir in the prevention of mother-to-child transmission of HBV in Africa. Cost effectiveness analysis is urgently needed to prove that targeted approach with HBV birth dose immunization among women infected with HBV would be cost effective compared to universal immunization coverage, specifically in the region where there is low HBV replication and also its impact on the HBV associated chronic liver diseases.

## Conclusion

In Togo, one out of 10 childbearing women were infected with HBV, but less than 2% of infant under 5 years old who received HBV immunization in the EPI were infected with HBV. With antenatal screening and targeted interventions, such as the introduction of Tenofovir in women and birth doses vaccination policies in babies, elimination of HBV in Togo could be achievable.

## Data Availability

The datasets used and/or analysed during the current study are available from the corresponding author on reasonable request.

## Abbreviations

95%CI: 95% confidence interval
aOR: Adjusted Odds Ratio
HBsAg: HBs Antigen
HBV: Hepatitis B virus
IQR: Interquartile range
MTCT: Mother-to-child-transmission
MSM: Men who have sex with men
OR: Odds Ratio
SSA: Sub-Saharan Africa
WHO: World Health Organization
